# Evaluating Short-Term and Long-Term Risks Associated with Renal Artery Stenosis Position and Severity: A Hemodynamic Study

**DOI:** 10.3390/bioengineering10091002

**Published:** 2023-08-24

**Authors:** Yawei Zhao, Yike Shi, Yusheng Jin, Yifan Cao, Hui Song, Lingfeng Chen, Fen Li, Xiaona Li, Weiyi Chen

**Affiliations:** 1 College of Biomedical Engineering, Taiyuan University of Technology, Taiyuan 030024, China; zyw213596@163.com (Y.Z.); syk10052000@163.com (Y.S.); yus15535219833@163.com (Y.J.); cyf7793495792021@163.com (Y.C.); lixiaona@tyut.edu.cn (X.L.); chenweiyi@tyut.edu.cn (W.C.); 2 College of Mechanical and Vehicle Engineering, Taiyuan University of Technology, Taiyuan 030024, China; songhui@tyut.edu.cn; 3 Institute of Applied Mechanics, Taiyuan University of Technology, Taiyuan 030024, China

**Keywords:** renal artery stenosis, computational fluid dynamics, hemodynamics, renal perfusion

## Abstract

*Background:* Moderate renal artery stenosis (50–70%) may lead to uncontrolled hypertension and eventually cause irreversible damage to renal function. However, the clinical criteria for interventional treatment are still ambiguous in this condition. This study investigated the impact of the position and degree of renal artery stenosis on hemodynamics near the renal artery to assess the short-term and long-term risks associated with this disease. *Methods:* Calculation models with different degrees of stenosis (50%, 60%, and 70%) located at different positions in the right renal artery were established based on the computed tomography angiography (CTA) of a personalized case. And computational fluid dynamics (CFD) was used to analyze hemodynamic surroundings near the renal artery. *Results:* As the degree of stenosis increases and the stenosis position is far away from the aorta, there is a decrease in renal perfusion. An analysis of the wall shear stress (WSS)-related parameters indicated areas near the renal artery (downstream of the stenosis and the entrance of the right renal artery) with potential long-term risks of thrombosis and inflammation. *Conclusion:* The position and degree of stenosis play a significant role in judging short-term risks associated with renal perfusion. Moreover, clinicians should consider not only short-term risks but also independent long-term risk factors, such as certain regions of 50% stenosis with adequate renal perfusion may necessitate prompt intervention.

## 1. Introduction

Renal artery stenosis (RAS) is a narrowing of the major arteries that supply blood to the kidneys [1]. Approximately 90% of RAS cases are attributed to atherosclerosis [2,3]. The prevalence of RAS rises with age and is particularly high among patients with conditions such as hypertension, coronary artery disease, and other forms of atherosclerotic disease [3]. The probability of atherosclerotic RAS in individuals over 65 years of age is 7% [4]. RAS occurs in 1–5% of patients with hypertension, 30% of patients with coronary artery disease, and up to 50% of elderly patients with extensive atherosclerotic disease [5,6,7,8]. RAS can lead to Goldblatt hypertension and reduced blood flow, potentially resulting in ischemic nephropathy and renal failure [1,9,10]. In addition, there have been reported associations between RAS and other arterial diseases [7,11,12]. Therefore, early diagnosis and evaluation of RAS is vital for preventing the occurrence and progression of renal events and malignant arterial disease.

Clinically, treatment is often determined according to the severity of RAS patients. The degree of stenosis diagnosed using angiography is often used to describe the severity of RAS, often distinguished by a 50% stenosis severity [13,14,15]. In cases of mild renal artery disease with a stenosis severity of less than 50%, conservative medical treatment is often sufficient, primarily focusing on hypertension control [15]. As the degree of stenosis progresses, it becomes challenging to effectively manage blood pressure, and the potential for irreversible damage to renal function increases [15,16]. When the degree of stenosis exceeds 70%, surgical intervention like stent implantation angioplasty is required to effectively address the condition [15,16]. Nevertheless, there is currently a lack of consensus regarding the necessity of emergency intervention for patients with moderate RAS (50–70%) as determined using angiography. Hemodynamic severity seems to be a key factor in judging whether surgical intervention is needed [13,15,16]. For instance, clinicians can invasively measure a patient’s renal flow reserve fraction (FFR). If the value is less than 0.8, indicating a high severity of hemodynamics, surgical treatment is generally used [15,16,17]. In addition, a resting translesional mean pressure gradient higher than 10 mmHg or a hyperemic peak systolic pressure gradient exceeding 20 mmHg also indicates a high severity of hemodynamic impairment, and it is advisable to consider surgical intervention [17]. However, the experience of and techniques employed by clinicians can influence the accuracy of the measurements and potentially contribute to unintended harm to patients. If numerical simulation can be utilized to evaluate the hemodynamic severity of RAS, it would offer clinicians additional references to establish an optimal surgical strategy to improve both short- and long-term outcomes.

In the short term, reduced renal perfusion due to RAS results in a temporary decrease in blood flow, which may lead to renal hypoxia and ischemia, gradually causing renal dysfunction and potentially progressing to chronic renal failure [10]. Therefore, renal perfusion can be considered a short-term risk indicator for assessing the complications of RAS. In the long term, RAS may significantly impact the flow dynamics near the renal artery, resulting in an unfavorable hemodynamic environment. The continuous presence of disturbed hemodynamics may promote the development of RAS and other arterial diseases such as thoracic aortic plaque [12]. Parameters related to wall shear stress (WSS), including time-averaged wall shear stress (TAWSS), oscillatory shear index (OSI), and relative residence time (RRT), provide a more comprehensive understanding of the hemodynamic environment. Physiological reactions within the blood vessels are closely linked to changes in hemodynamic parameters. Research has demonstrated that abnormally high shear conditions are correlated with endothelial damage, increasing the risk of local thrombogenic and, subsequently, leading to lumen obstruction [18]. High OSI values and prolonged RRT are widely recognized as factors that contribute to thrombosis by promoting platelet aggregation, activating platelets, and prolonging the presence of procoagulant microparticles, thus increasing the risk of plaque formation [19,20,21]. These factors collectively increase the propensity for thrombus. Consequently, these parameters can be employed as valuable tools to assess the long-term risks associated with arterial diseases [22,23]. By understanding the short-term and long-term risks of RAS, clinicians can better evaluate the necessity of interventional treatments for moderate RAS.

The morphology of blood vessels plays a crucial role in alterations in the hemodynamic environment. Previously, researchers have used idealized models to simulate the effects of stenosis morphology (such as stenosis severity, stenosis length, stenosis symmetry, stenosis eccentricity, and renal branching angle) on hemodynamics [10,16,24,25]. These studies show that the degree of stenosis significantly influences alterations in the hemodynamic environment. However, few investigators have considered the effect of stenosis position using personalized models to assess the hemodynamics of moderate stenosis. By gaining a comprehensive understanding of the short-term and long-term injury mechanisms caused by the position and degree of stenosis, it may help clinicians to tailor interventions to individual patients.

In this study, computational fluid dynamics (CFD) numerical simulation was carried out based on an individualized model to study the hemodynamic effects of the position and degree of RAS. As indicated by our study, the position and degree of stenosis have a significant effect on the hemodynamic severity of RAS. When assessing the severity of hemodynamics, the comprehensive long-term risks in addition to short-term risk factors like renal perfusion should be taken into consideration by clinicians. The findings of this study can serve as a valuable reference for clinicians when assessing the treatment approach for moderate renal artery stenosis.

## 2. Materials and Methods

### 2.1. Geometry Modeling

**Figure 1:**
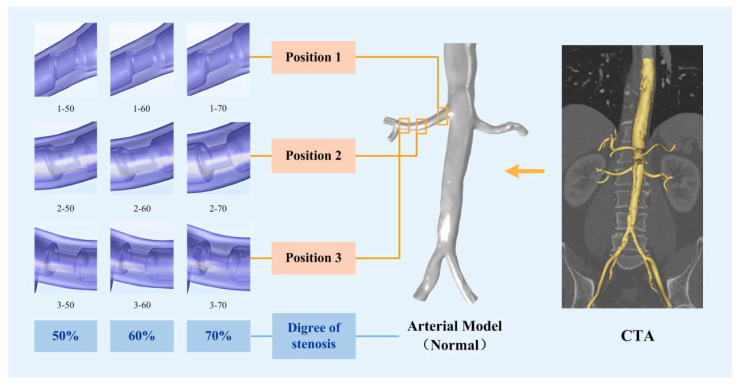
Normal and stenosis model diagram.

In this study, a case with preferable abdominal aortic morphology was selected. Based on its computed tomography angiography (CTA), a three-dimensional model of the abdominal aorta was reconstructed and optimized using Mimics 19.0 (Materialise, Plymouth, MI, USA). Then, models with different degrees of stenosis at three positions were constructed. As shown in Figure 1, the proximal, middle, and distal ends of the entrance of the right renal artery correspond to positions 1, 2, and 3, respectively. The degree of stenosis is defined as the ratio of the area before and after stenosis on the cross section ($SD=\frac{A_{1}-A_{2}}{A_{1}}$, where SD is the degree of stenosis; A_1_ is the lumen area of the renal artery before stenosis; and A_2_ is the lumen area of the renal artery after stenosis) [16]. The model is marked as position-severity (e.g., 1-50).

### 2.2. Governing Equations

Blood is assumed to be incompressible, homogenous, and non-Newtonian fluid in the simulation, which is based on the three-dimensional incompressible Navier–Stokes equations and the continuity equations:

(1) $$\rho \left(\frac{\partial \vec{v}}{\partial t}+\left(\vec{v}\cdot \nabla \right)\vec{v}\right)=-\nabla p+\nabla \tau$$

(2) $$\nabla \cdot \vec{v}=0,$$

where ρ is the blood density (1050 kg/m^3^), $\vec{v}$ is the fluid velocity vector, p represents the pressure, and τ is the stress tensor.

### 2.3. Boundary Condition and Calculation Methods

**Figure 2:**
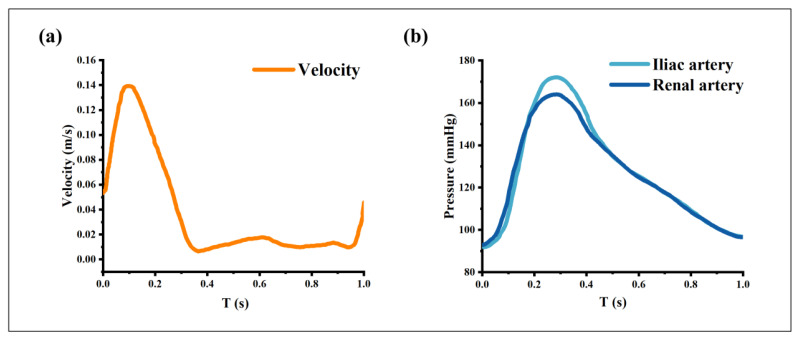
Boundary conditions. (**a**) Inlet velocity waveform, (**b**) Outlet pressure waveforms.

The computation involves two steps: a steady flow simulation followed by a pulsatile flow simulation. The solution of the steady simulation provides reasonable initial values for the unsteady pulsatile simulation. The velocity and pressure conditions used for the steady flow are those of the pulsatile flow at time = 0, namely a mean inlet velocity of approximately 0.044 m/s, and outlet pressures of 12,631 Pa and 12,646 Pa for the renal artery and iliac artery, respectively. For the pulsatile flow simulation, the velocity waveform was imposed as the inlet boundary condition (Figure 2a) [26]. Different time-dependent pressure conditions were applied at the renal artery and iliac artery outlets (Figure 2b) [27]. Additionally, the arterial walls were assumed to be no-slip and rigid.

The commercial software COMSOL Multiphysics (V5.5) was used for numerical simulation to investigate the flow characteristics. To ensure a complete flow, the inlet and outlet were extended to five times the diameter. The simulated cardiac cycle lasted for 1.0 s, encompassing a total of five cycles, with a time step of 0.01 s. After grid independence verification, the final number of grids was about 401,740. The analysis focused on the results obtained from the final cycle.

### 2.4. Related Indicators

For all the models, perfusion ($\frac{Q_{n}}{Q_{0}}$, where Q_n_ is the flow of each outlet, with *n* = 1 to 4, and Q_0_ is the flow of the inlet) and hemodynamic parameters based on wall shear stress, including TAWSS, OSI, and RRT, were evaluated. The mathematical expressions for these parameters are presented below.

TAWSS measures the time-averaged wall shear stress throughout the cardiac cycle, and the formula is as follows:

(3) $$TAWSS=\frac{1}{T}\int_{0}^{T}\left|WSS\left(s,t\right)\right|dt,$$

where *T* is the cycle period, and *WSS*(*s*,*t*) describes the frictional force exerted by blood flow on the vessel wall per unit area. This formula calculates the average wall shear stress experienced by the vessel wall over the entire duration of the cardiac cycle. It provides insights into the overall hemodynamic forces acting on the vessel during the cycle.

OSI is a parameter used to quantify the directional changes in the WSS vector during the pulsation period. It is expressed as a numerical value ranging from 0 to 1. A value closer to 1 indicates greater oscillation and directional changes in the WSS vector, suggesting a more disturbed and non-unidirectional flow pattern. Conversely, a value closer to 0 indicates a more stable and predominantly unidirectional flow pattern. The formula for calculating OSI is as follows [26]:

(4) $$OSI=\frac{1}{2}\left[1-\left(\frac{\left|\int_{0}^{T}WSS\left(s,t\right)dt\right|}{\int_{0}^{T}\left|WSS\left(s,t\right)\right|dt}\right)\right]$$

RRT is a parameter used to evaluate the residence time of particles or fluid elements moving within a flow field. It provides information about the exposure of these particles to the flow conditions. A higher RRT value suggests a longer residence time in regions with low wall shear stress, potentially indicating areas of flow stagnation or disturbed flow. The formula for calculating RRT is as follows [28]:

(5) $$RRT=\frac{1}{\left(1-2\times OSI\right)\times TAWSS}$$

## 3. Results

### 3.1. Flow Pattern

**Figure 3:**
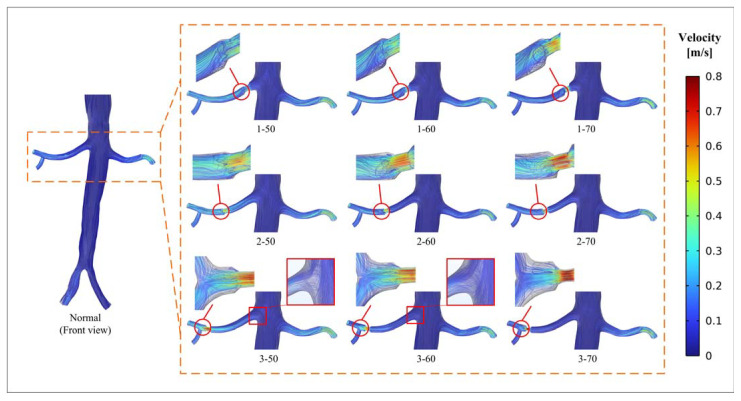
Velocity distributions at the peak pressure (front view).

Figure 3 illustrates the characteristics of velocity streamlines at the peak pressure. The aorta exhibits a low velocity, while a relatively higher velocity is observed in the renal artery, with the peak velocity occurring at the stenotic segment. The peak velocity increases with an increase in stenosis severity. As the distance between the stenotic segment and the aorta increases, there tends to be an associated increase in the peak velocity at the stenosis. A vortex appears in the narrow downstream. With the increase in stenosis severity, the vortex becomes more obvious, and the size of the recirculation zone expands. An obvious recirculation zone appears after the simulation of stenosis in model 3-70 (position 3 with 70% stenosis). Additionally, flow separation is observed at the region between the aorta and the renal artery, and a vortex circulation is generated within this separation zone.

### 3.2. Renal Perfusion

**Figure 4:**
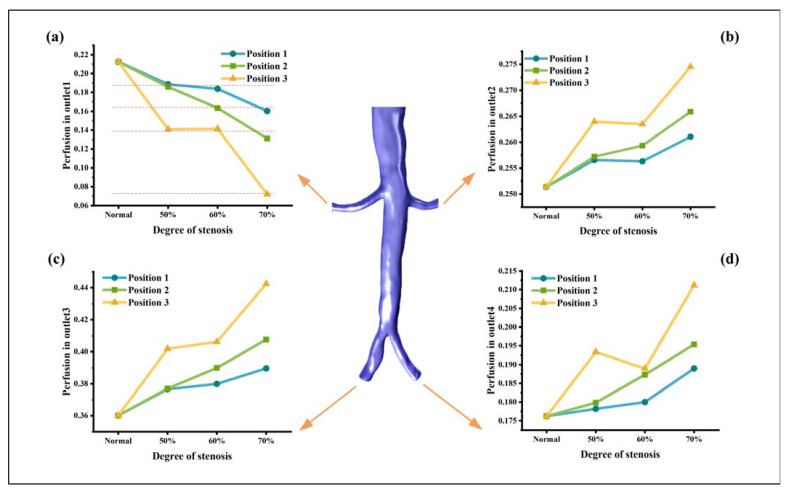
The perfusion of each outlet of the model. (**a**) Right renal artery, (**b**) Left renal artery, (**c**) Right iliac artery, (**d**) Left iliac artery.

In Figure 4, the perfusion (as defined in the Materials and Methods Section) of each outlet is illustrated. After the occurrence of stenosis, a reduction in perfusion can be observed in the affected renal artery, while an increase in perfusion is seen in the contralateral renal artery. The increasing trend of the stenosis side is similar to the decreasing trend of the contralateral side. Figure 4a demonstrates that as the degree of stenosis increases, the perfusion gradually decreases. Specifically, there is a gradual decline in perfusion from 50% stenosis to 60% stenosis, followed by a sharp decrease from 60% stenosis to 70% stenosis. At position 3, the perfusion experiences the most significant reduction, with a decline rate of 66.04%. Furthermore, the perfusion decreases as the stenosis position moves further away from the aorta, with stenosis at position 3 having the most pronounced impact on the perfusion. A horizontal comparison shows that 50% stenosis and 60% stenosis at position 1, as well as 50% stenosis at position 2, are associated with comparable perfusion, with a value of about 18.7%. When 60% stenosis occurs at position 2 and 70% stenosis occurs at position 1, the perfusion of the affected side of the renal artery is similar and the value is about 16.2%. The combination of 50% and 60% stenosis at position 3 and 70% stenosis at position 2 results in comparable perfusion, and the percentage is about 13.9%. The renal artery with 70% stenosis at position 3 exhibits the lowest perfusion, showing a value of approximately 7.2%.

### 3.3. WSS-Related Parameters

#### 3.3.1. TAWSS

**Figure 5:**
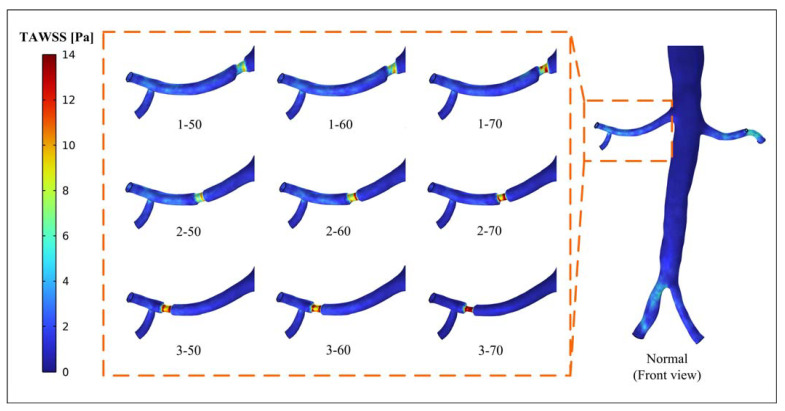
Distribution of time-averaged wall shear stress (TAWSS; front view).

Figure 5 demonstrates the distribution of TAWSS in the right renal artery. The figure shows that the narrow segment of the artery exhibits elevated TAWSS values. As the degree of stenosis increases, the extent of high TAWSS expands. Similarly, when the stenosis position is far away from the aorta, the high TAWSS area and value of the stenotic segment increase. In model 3-70, the narrow segment is filled with abnormally high TAWSS.

#### 3.3.2. OSI

**Figure 6:**
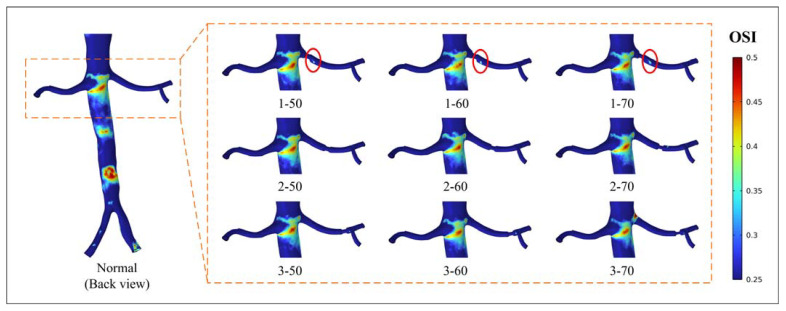
Distribution of oscillatory shear index (OSI) > 0.25 (back view).

**Figure 7:**
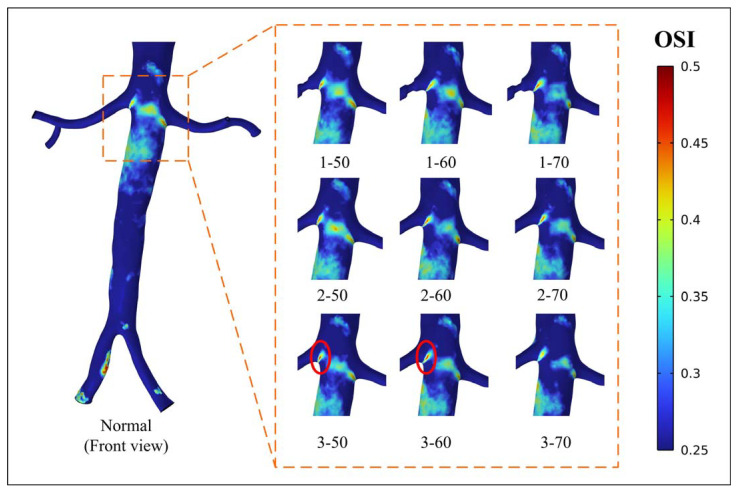
Distribution of OSI > 0.25 (front view).

Figure 6 and Figure 7 display the regional distribution of OSI exceeding 0.25. Figure 6 illustrates the occurrence of localized high OSI values downstream of the narrow segment. The distribution of high OSI is predominantly observed in all models, except for models 2-50 and 2-60. The most significant high OSI is observed at position 1. In Figure 7, a notable distribution of high OSI is observed in the abdominal aorta near the entrance of the renal artery. The appearance of stenosis changes the OSI peak at the entrance of the renal artery. In models 3-50 and 3-60, the abnormally high peak of OSI is found at the entrance of the right renal artery, and the value is 0.4952 and 0.4972, respectively.

#### 3.3.3. RRT

**Figure 8:**
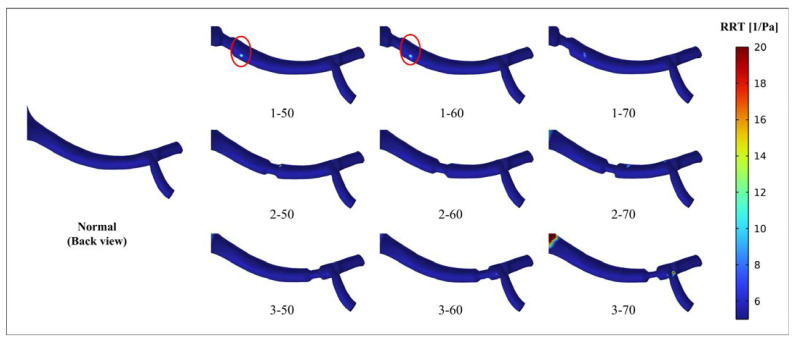
Distribution of relative residence time (RRT) > 5 Pa^−1^ (renal artery in back view).

**Figure 9:**
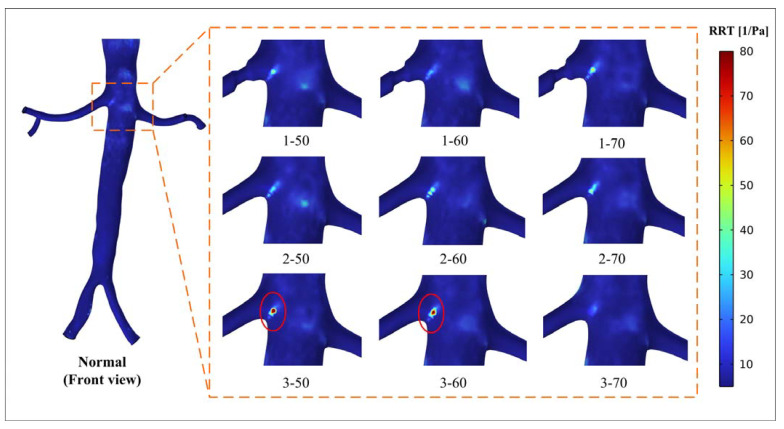
Distribution of RRT > 5 Pa^−1^ (aorta in front view).

Figure 8 and Figure 9 show the RRT distribution of the renal artery and the local aorta. Figure 8 illustrates the distribution of RRT greater than 5 Pa^−1^ in the renal artery. The results indicate the presence of a high RRT area downstream of the narrow segment in models 1-50 and 2-50, which corresponds to the high OSI area observed. As shown in Figure 9, the regional distribution of RRT exceeds 5 Pa^−1^ in the abdominal aorta near the entrance of the renal artery. After the occurrence of stenosis, there is an increase in RRT at the entrance of the affected renal artery. Particularly in models 3-50 and 3-60, there are abnormally high peaks of RRT values, as indicated by values of 347.14 Pa^−1^ and 568.02 Pa^−1^, respectively. This observation aligns with the distribution of the abnormally high OSI previously shown in Figure 6 and Figure 7.

## 4. Discussion

Currently, the clinical criteria for determining the need for interventional treatment in cases of moderate renal artery stenosis (50–70%) remain unclear. Hemodynamic studies can play a crucial role in providing additional information to clinicians for making decisions. From the perspective of short-term risks, the narrowing of the renal artery results in an immediate reduction in blood flow, leading to renal hypoperfusion. This may cause renal ischemia and inadequate oxygen supply, ultimately resulting in chronic damage to renal function [9,10]. When evaluating the long-term risks, specific hemodynamic parameters, such as TAWSS, OSI, and RRT, can provide insights into potential risks. These parameters offer valuable information regarding hemodynamic characteristics and can be indicative of certain risks. In this study, a personalized model was utilized to investigate the hemodynamic effects of position and degree of RAS. Critical factors, including renal perfusion, and hemodynamic parameters in the renal artery and near the entrance of the renal artery, were taken into consideration. The results of this study can provide clinicians with short-term and long-term risk information for moderate RAS.

The results showed that as the stenosis severity increased, the blood supply to the affected kidney decreased, while the blood supply to the contralateral kidney increased. This imbalance in renal perfusion may lead to impaired renal function, primarily due to abnormal perfusion in the resting state [29,30]. Insufficient blood supply to the affected kidneys in RAS patients results in a gradual loss of renal function [2,29,30,31]. To compensate for the reduced perfusion in the kidney with stenosis, the unaffected kidney undergoes an elevation in blood pressure and perfusion [31]. While this compensatory mechanism helps maintain blood flow in the unaffected kidney, it also places an additional burden on that kidney, potentially leading to damage over time [31]. It is noteworthy that 70% stenosis resulted in a significant decrease in renal perfusion, which is consistent with the clinical criteria for selecting a 70% stenosis threshold for interventional therapy.

However, renal perfusion is not only related to the degree of stenosis but also to the position of stenosis. As the stenosis position moves away from the renal entrance, the stenosis severity becomes more influential on the perfusion. It can be observed in Figure 4a that the perfusion in model 1-70 (position 1 with 70% stenosis) was even higher than that in models 3-50 and 3-60. By establishing the assumption that stenosis patterns with similar perfusion carry an equivalent risk, it becomes possible to evaluate the short-term risk associated with RAS. As the risk level increases, the short-term risk of RAS follows a corresponding escalation. By considering perfusion and its correlation with risk levels, clinicians can make informed decisions regarding the management and treatment of RAS patients, thereby optimizing their care and minimizing the potential short-term complications associated with this condition.

Hemodynamic parameters, such as TAWSS, OSI, and RRT, can describe the long-term potential risk of arterial disease. By conducting an analysis of these parameters, a more comprehensive understanding of the physiological response between blood flow and the vascular wall can be obtained. This analysis enables the identification of regions characterized by disturbed flow, which may indicate regions of heightened vulnerability to arterial diseases [32]. The combination of high TAWSS and a vortex will activate platelets, and activated platelets have the potential for blood clot formation in regions characterized by elevated OSI and RRT [33,34]. In this study, stenosis occurring in the renal artery led to alterations in the flow pattern, creating an undesirable hemodynamic environment near the stenotic segment. The results showed that the stenotic segment of the renal artery showed a high TAWSS region, and the flow field downstream of the stenotic part produced a vortex, which was accompanied by high OSI and high RRT regions (Figure 5, Figure 6, and Figure 8). The presence of a distinct recirculation area formed by the vortex following stenosis suggests the entrapment of platelet activation within these stagnant regions. This phenomenon promotes the transport of activated platelets and their subsequent adhesion to the vessel wall, increasing the long-term risk of thrombosis [35]. Therefore, although the perfusion shows little variation between models 1-50 and 1-60, the presence of regions with high OSI and RRT suggests a risk of thrombosis. It implies that interventional treatment should be considered in these scenarios based on a long-term thrombotic risk assessment.

The correlation between high OSI, RRT, and thrombosis formation has been widely acknowledged in the literature. Elevated OSI and RRT can promote platelet aggregation, activate platelets, and extend the residence time of procoagulant microparticles, thereby increasing the likelihood of thrombosis development [19,20,21]. The personalized model used in this study showed a specific hemodynamic environment due to its unique anatomy. The existence and morphology of stenosis in the renal artery affect the surrounding flow field, thereby altering the specific hemodynamic conditions. The results indicated that all models displayed a concentrated distribution of high OSI in the region of the abdominal aorta near the renal artery (Figure 6 and Figure 7). This occurrence can be attributed to the presence of a vortex resulting from the disparity in height between the two renal arteries and the intricate geometry of the inlet of the renal artery. The flow field in this particular region is influenced by the position and degree of stenosis. Importantly, the region near the entrance of the right renal artery exhibited peak values for both RRT and OSI in models 3-50 and 3-60 (Figure 7 and Figure 9). When there is a high OSI, it indicates a flow field with significant oscillation in WSS. This irregular flow pattern results in uneven distribution and localized intensification of blood flow shear stress, which triggers platelet activation and facilitates platelet aggregation. Meanwhile, high RRT prolongs the residence time of procoagulant particles in the blood. The interplay between high OSI and high RRT synergistically enhances platelet aggregation and intensifies the interaction between platelets and endothelial cells, ultimately escalating the risk of thrombosis formation [19,20,21]. Therefore, there is an elevated risk of thrombosis and inflammation in cases of 50% and 60% stenosis near the entrance of the renal artery at position 3. Considering the long-term implications regarding thrombotic events and inflammation, addressing these conditions through interventions becomes all the more imperative.

There are certain limitations to this study. Firstly, this research exclusively relies on a personalized model, and the reliance on one model introduces an inherent structural bias that may influence the obtained results to some extent. The individual variations in anatomy and hemodynamics may not be fully captured by the model, leading to potential inaccuracies. Secondly, the stenosis considered in this study assumes a typical central stenosis scenario. However, in actual cases, unilateral stenosis is more prevalent. In addition, the stenosis is located on the higher side of the renal artery. When the stenosis is located on the lower side of the renal artery, whether it will show the same trend as described in this paper is worthy of further discussion. Thirdly, the application of identical pressure boundary conditions to each outlet of the renal artery and iliac artery deviates from the actual physiological conditions. In reality, there are variations in pressure and flow at different outlets, and accounting for these differences could provide a more accurate representation of hemodynamics. Furthermore, it should be noted that the results of simulations are influenced by boundary conditions. Scholars have been exploring different methods to incorporate realistic boundary conditions, aiming to improve the accuracy of simulations. Consequently, there may be disparities between simulation outcomes and clinical measurements. Finally, the assumption of a rigid and non-slip vascular wall may not fully reflect real-life physiological responses. The arterial walls in the human body are elastic and exhibit certain degrees of movement and deformation, which can influence the flow patterns and distribution of shear stress. Incorporating more realistic wall properties into the model could enhance the accuracy of the results.

## 5. Conclusions

**Figure 10:**
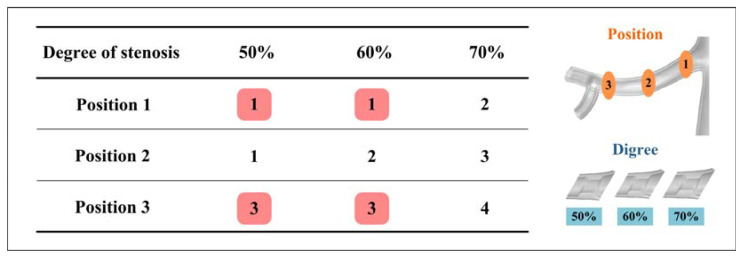
Short-term and long-term risk diagram of renal artery stenosis (the number represents the short-term risk level associated with renal perfusion; the red area represents the long-term risk associated with the WSS-related parameters).

The position and degree of stenosis have a significant effect on the hemodynamic severity of RAS. By utilizing computational fluid dynamics, it becomes possible to consider and quantify the short-term and long-term risks associated with moderate RAS. As shown in Figure 10, the number represents the short-term risk level associated with renal perfusion, and the parts highlighted in red represent the long-term risk associated with the WSS-related parameters. The findings provide clinicians with essential short-term and long-term risk information concerning the position of moderate stenosis, aiding in more informed decision making.

## Data Availability

Not applicable.
